# CHOT INDUSTRY PARTNER

**DOI:** 10.1093/geroni/igz038.1549

**Published:** 2019-11-08

**Authors:** Kenneth Cochran

**Affiliations:** University of Alabama at Birmingham, UAB, Alabama, United States

## Abstract

The third speaker will be the industry partner representative, Kenneth Cochran, President and CEO of Opelousas General Health System. Here we will hear from industry partners about what drew them to this program of inter-collaboration; the benefits they have received, and what was required of them to participate. This will be helpful as it will provide an opportunity to have a first-hand account about the value that practitioners find in this program as it relates to testing interventions, developing new systems, and improving financial performance and/or quality. Kenneth will speak to a particular project at Opelousas and describe entire process. Kenneth will highlight the challenges and opportunities that come from partnering with an academic institution. Also, how CHOT brings together industry partners who are facing the same challenges and how they learn and adopt best practices from each other. This should further illustrate the value that academic/industry collaboration can bring.

